# Structural basis of RNA polymerase II transcription on the histone H3–H4 octasome

**DOI:** 10.1016/j.jbc.2026.111340

**Published:** 2026-03-04

**Authors:** Cheng-Han Ho, Kayo Nozawa, Masahiro Nishimura, Mayuko Oi, Tomoya Kujirai, Mitsuo Ogasawara, Haruhiko Ehara, Shun-ichi Sekine, Yoshimasa Takizawa, Hitoshi Kurumizaka

**Affiliations:** 1 Laboratory of Chromatin Structure and Function, Institute for Quantitative Biosciences, The University of Tokyo, Tokyo, Japan; 2 Department of Biological Sciences, Graduate School of Science, The University of Tokyo, Tokyo, Japan; 3 Laboratory for Transcription Structural Biology, RIKEN Center for Integrative Medical Sciences, Yokohama, Japan

**Keywords:** RNA polymerase II, chromatin, nucleosome, H3–H4 octasome, subnucleosome, histone, cryo-EM, transcription

## Abstract

The histone H3–H4 octasome is a nucleosome-like particle in which two DNA gyres are wrapped around each histone (H3–H4)_2_ tetramer disk, forming a clamshell-like configuration. In the present study, we performed *in vitro* RNA polymerase II (RNAPII) transcription assays with the H3–H4 octasome and found that RNAPII transcribed the H3–H4 octasome more efficiently than the nucleosome. RNAPII paused at only one position, superhelical location (SHL(-4)) in the H3–H4 octasome, in contrast to pausing at the SHL(-5), SHL(-2), and SHL(-1) positions in the nucleosome. Cryo-EM analysis revealed that two (H3–H4)_2_ tetramer disks are retained when the RNAPII paused at the SHL(-4) position of the H3–H4 octasome. However, when RNAPII reached the SHL(-0.5) position, five base pairs before the dyad position of the H3–H4 octasome, the proximal (H3–H4)_2_ tetramer was disassembled, but the distal (H3–H4)_2_ tetramer still remained on the DNA. Therefore, RNAPII efficiently transcribes the H3–H4 octasome by stepwise (H3–H4)_2_ tetramer disassembly.

In eukaryotic cells, genomic DNA is packed into the cell nucleus by chromatin, primarily through the formation of a fundamental unit called the nucleosome (1). Each nucleosome consists of DNA wrapped around a histone octamer, a specialized structure composed of two copies each of the core histones H2A, H2B, H3, and H4 (2) (Fig. 1, *A* and *C*). The locations of the DNA wrapped around the nucleosome are referred to as superhelical locations (SHLs) (2, 3) (Fig. 1*A*). At the dyad axis of the nucleosome, the minor groove of the DNA facing outward from the histone core is defined as SHL(0). Subsequent outward-facing minor grooves are referred to as SHL ±1 to 7, with the plus and minus signs indicating the direction. The distance between each SHL roughly corresponds to 10 base pairs of DNA.

**Figure 1:**
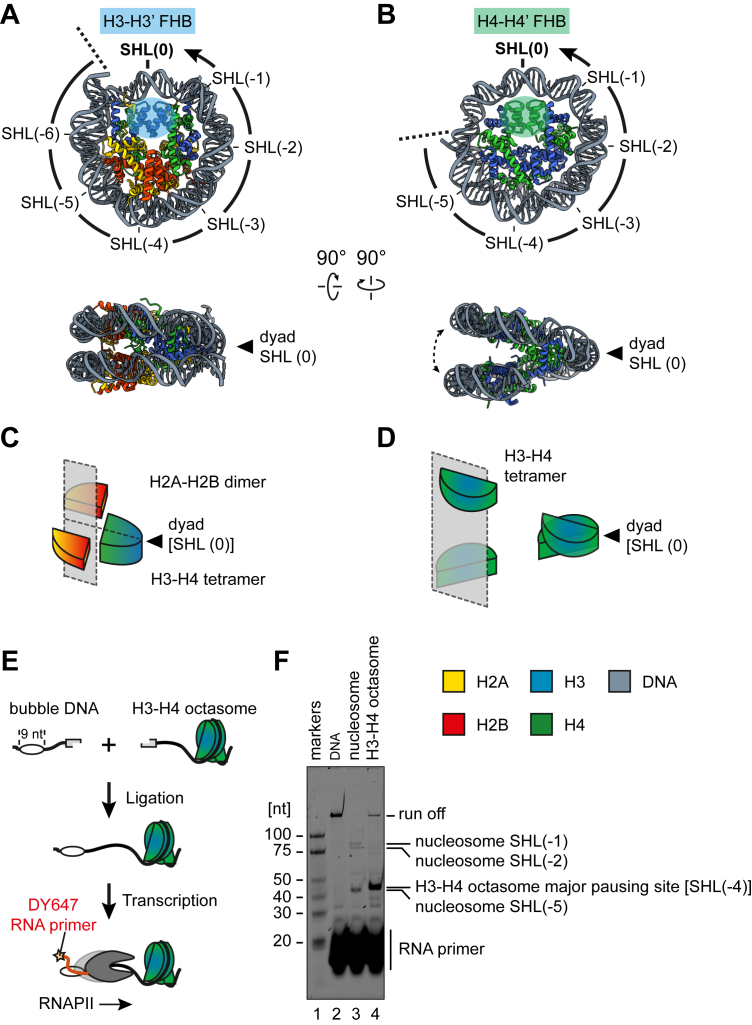
**Transcription profile of the H3–H4 octasome.***A, ribbon model* of the nucleosome (PDB ID: 3LZ0), with the superhelical locations (SHLs) and the H3–H3′ four-helix bundle (FHB) depicted. *B, ribbon model* of the H3–H4 octasome (closed form, PDB ID: 7X57), with the SHLs and the H4–H4′ FHB depicted. *C,* schematic representation showing the relative positions of the H2A–H2B dimers and the (H3–H4)_2_ tetramer in the nucleosome. *D,* schematic representation showing the relative positions of the (H3–H4)_2_ tetramers in the H3–H4 octasome. *E,* schematic representation of the transcription assay. *F,* denaturing gel electrophoresis analysis of the RNA products from the transcription assay with RNAPII and DNA, the nucleosome, or the H3–H4 octasome. The assay was repeated three times independently, and consistent results were obtained (Fig. S2). RNAPII, RNA polymerase II.

Although the nucleosome contributes to packaging DNA in the nucleus, it also serves as a functional machinery for many biological processes, such as replication (4), recombination (5), repair (6, 7), and transcription (8, 9, 10, 11, 12, 13, 14, 15, 16, 17, 18), as revealed by cryo-EM. For transcription elongation, the DNA wrapped around the nucleosome is gradually peeled off by RNA polymerase II (RNAPII), while pausing at several SHLs: SHL(-6), SHL(-5), SHL(-2), and SHL(-1) (8, 10) (Fig. 1*A*). Each of those SHLs is a major DNA–histone interaction site, suggesting that the DNA–histone interaction hinders the process of DNA peeling by RNAPII. During the transcription of the nucleosome, the histone core remains octameric until RNAPII reaches SHL(-1). The nucleosome is then disassembled after the RNAPII passes through the SHL(0) position and reassembled behind the RNAPII (15).

The nucleosome is a highly dynamic structure and may undergo transitions through various intermediate states, also known as subnucleosomal forms (19, 20, 21). Subnucleosomes are histone–DNA complexes with different stoichiometries compared with the nucleosome. The H3–H4 octasome is a subnucleosome, in which the DNA is wrapped around the H3–H4 octamer with two (H3–H4)_2_ tetramers (22, 23, 24, 25) (Fig. 1, *B* and *D*). Early EM studies and a previous atomic force microscopy analysis demonstrated that the H3–H4 octasome forms a bead-like structure with dimensions similar to that of the nucleosome (26, 27). Our recent cryo-EM analysis further revealed the detailed structures of the H3–H4 octasome (25). As in the nucleosome, the DNA is left-handedly wrapped around the H3–H4 histone octamer, but significant differences exist between the H3–H4 octasome and the nucleosome. First, the histone positioning relative to SHL(0) is different. In the nucleosome, the H3–H3′ four-helix bundle (FHB) (3), the interaction site of the two H3 protomers in the (H3–H4)_2_ tetramer, is located at SHL(0) (Fig. 1*A*). In contrast, in the H3–H4 octasome, the H3–H3′ FHBs are located at the SHL(±3) positions, flanking the SHL(0) (dyad), and a novel H4–H4′ FHB is present at SHL(0) (Fig. 1*B*). Second, approximately 120 base pairs of DNA are wrapped around the H3–H4 octasome, which is shorter than the 145 to 147 base pairs of DNA wrapped in the nucleosome (2) (Fig. 1, *A* and *B*). Third, two (H3–H4)_2_ tetramers of the H3–H4 octasome form a clamshell-like conformation, which is flexible and expands the distance between two DNA gyres compared with the nucleosome (Fig. 1, *B* and *D*).

The unique features of the H3–H4 octasome, particularly in terms of histone positioning, DNA wrapping length, and clamshell-like structural flexibility, suggest potential implications for distinct transcriptional regulation mechanisms. To study the transcription profile of the H3–H4 octasome by RNAPII, in the present study, we performed *in vitro* nucleosome transcription experiments coupled with cryo-EM analysis.

## Results

### The transcription profile of the H3–H4 octasome is remarkably different from that of the nucleosome

To investigate the transcription process of the H3–H4 octasome, we first prepared it with a previously reported template DNA sequence for the transcription assay (8) (Fig. S1, *A* and *B*). The template DNA, which contains a nine-base mismatch, allows the RNA primer to bind (Fig. 1*E*). This RNA primer is extended by transcription elongation with RNAPII and thus serves as an indicator of the RNAPII pausing position on the transcribed template. We purified RNAPII from the yeast *Komagataella phaffii* and prepared the elongation factor TFIIS as a recombinant protein, as previously described (28). Using the prepared H3–H4 octasome template, RNAPII, and TFIIS, we performed a transcription assay *in vitro*. The reaction mixtures were analyzed by denaturing PAGE, and the transcribed RNAs were detected. In this assay, the template DNA and the template nucleosome were also included for comparison.

In the transcription assay with the naked DNA template, RNAPII pausing was barely observed, and most of the RNAs were run-off transcription products (Fig. 1*F*, lane 2). Consistent with the previous studies, with the nucleosome template, RNAPII pauses at SHL(-5), SHL(-2), and SHL(-1) were observed, and the run-off transcript was barely detected (Fig. 1*F*, lane 3) (8). Interestingly, with the H3–H4 octasome template, an intense but somewhat slower migrating band was observed near the nucleosomal SHL(-5) pausing site (Fig. 1*F*, lane 4). We hypothesized that the H3–H4 octasome may be positioned similarly to the nucleosome in the template DNA. According to the hypothesis, the major pausing site of the H3–H4 octasome would correspond to a position after SHL(-5), possibly SHL(-4). The run-off transcript was detected with the H3–H4 octasome but not the nucleosome, suggesting that RNAPII can transcribe the H3–H4 octasome more efficiently than the nucleosome (Fig. 1*F*, lane 4). No other noticeable bands were detected between the band just above the SHL(-5) band and the run-off band. This suggests that, unlike nucleosomal transcription, which has several pausing sites, RNAPII pauses only at one major site located near the H3–H4 octasome entry site, which may correspond to SHL(-4).

### Cryo-EM structure of RNAPII paused on the SHL(-4) position of the H3–H4 octasome

To clarify the mechanism of RNAPII pausing on the H3–H4 octasome, we performed cryo-EM analysis. The template DNA used in this cryo-EM analysis was modified so that RNAPII would pause at SHL(-0.5) (5 base pairs before the SHL(0) position) after passing the major SHL(-4) pausing site (Fig. S3*A*). Under the experimental setup, RNAPII pauses at the SHL(-0.5) site after overcoming the major natural pausing site, which may provide another snapshot structure during the transcription process by cryo-EM with one sample preparation (Fig. S3, *B* and *C*). This allows us to trace the structural transition during the RNAPII passage through the H3–H4 octasome. Using this experimental setup, we purified the RNAPII–H3–H4 octasome complex by sucrose/glutaraldehyde gradient ultracentrifugation (GraFix) method (Fig. S3*D*) and proceeded to the cryo-EM single-particle analysis.

**Figure 2:**
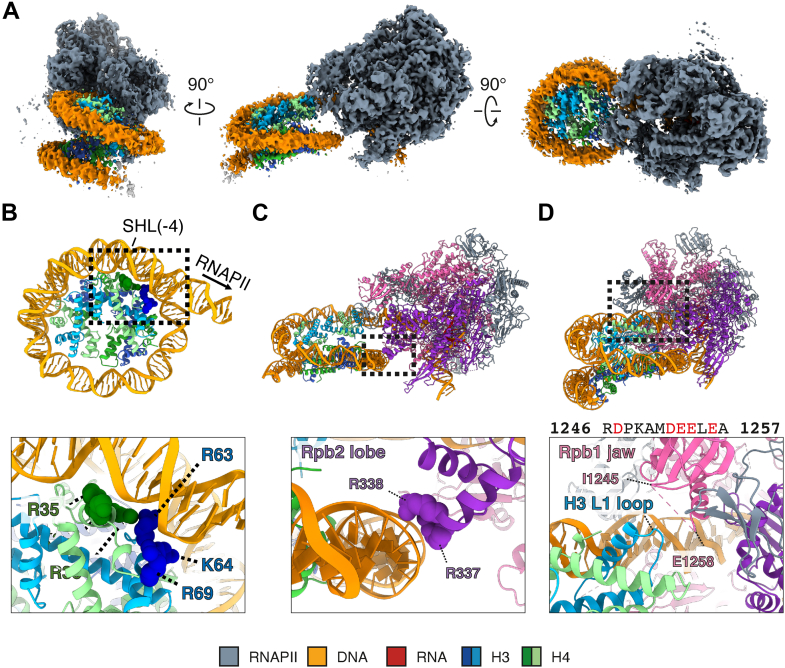
**Cryo-EM structure of the RNAPII-H3–H4 octasome (SHL[-4]).***A,* cryo-EM density maps of the RNAPII–H3–H4 octasome (SHL[-4]). Three different views of the same map are shown. *B,* interactions between DNA and histones when RNAPII pauses at SHL(-4) in the H3–H4 octasome. The *lower panel* shows a close-up view of the boxed region in the *upper panel*. The histone residues of H3 (*blue*) and H4 (*green*) that interact with DNA are indicated. *C,* interaction between RNAPII subunit Rpb2 (*purple*) and H3–H4 octasomal DNA (*orange*). The *lower panel* shows a close-up view of the boxed region in the *upper panel*. The arginine residues of Rpb2 involved in the interaction are indicated. *D,* possible interaction between RNAPII subunit Rpb1 (*pink*) and the H3 L1 loop (*blue*). The *lower panel* shows a close-up view of the boxed region in the *upper panel*. The amino acid residues between I1245 and E1258 of the Rbp1 jaw, which are invisible in this structure, are shown above the *lower panel*. Several basic residues in this region could interact with the H3 L1 loop, which contains several acidic residues. RNAPII, RNA polymerase II; SHL, superhelical location.

We first obtained the high-resolution cryo-EM structure of RNAPII paused at the major natural pausing site of the H3–H4 octasome, which indeed corresponds to the SHL(-4) position (Figs. 2*A*, S4, and S5). RNAPII collides with the H3–H4 octasome at the major natural SHL(-4) pausing site, without affecting the histone containing two (H3–H4)_2_ tetramers. In nucleosome transcription, RNAPII pauses at the major contact sites between DNA and core histones (8). Consistently, in the H3–H4 octasome, RNAPII pauses on the DNA, contacting six basic amino acid residues (H3R63, H3K64, H3R69, H4K31, H4R35, and H4R36) around the SHL(-4) position (Fig. 2*B*). Interestingly, these residues also induce RNAPII pausing at the SHL(-1) position in the nucleosome (8).

In the RNAPII–H3–H4 octasome complex paused at the SHL(-4) position, two arginine residues, R337 and R338, in the lobe of the RNAPII Rpb2 subunit are near the DNA wrapped around the H3–H4 octamer (Fig. 2*C*). In addition, the RNAPII subunit, Rpb1, may contact the H3 L1 loop (Fig. 2*D*). The R1246–A1257 region of the Rpb1 jaw contains several acidic residues, which may interact with the H3 L1 loop *via* the basic residues K79 and R83, although the Rpb1 R1246–A1257 region is not visible, probably because of its flexibility or multiple conformations (Fig. 2*D*, *lower panel*). These histone–DNA contacts at the SHL(-4) position, the RNAPII–DNA interaction, and the RNAPII–histone interaction may promote the RNAPII pausing at the SHL(-4) position of the H3–H4 octasome.

### An (H3–H4)_2_ tetramer dissociates after RNAPII overcomes the SHL(-4) barrier

In the template DNA used for cryo-EM analysis, a “T” site is newly added at position 74 base pairs from the transcription priming site (presumably SHL(-0.5) position) of the H3–H4 octasome (Fig. S3*A*). A “TTTT” site also exists 13 base pairs downstream of the inserted “T” site. *In vitro* H3–H4 octasome transcription reactions were then conducted in the presence of 3′-dATP, instead of ATP. A single “T” insertion may be too weak to completely stop the RNAPII progression; therefore, the RNA product that originated from RNAPII stalled at the “TTTT” site was also detected (Fig. S3, *B* and *C*).

Using the cryo-EM sample prepared by the GraFix method from the previous section (Fig. S3*D*), we also determined the cryo-EM structure of RNAPII intentionally paused at the SHL(-0.5) position of the H3–H4 octasome, which corresponds to the inserted “T” site. Note that we could not obtain the structure of RNAPII paused at the “TTTT” site corresponding to the SHL(+1) position (Figs. S4 and S6). It is possible that when transcription has reached after passing the dyad of the H3–H4 octasome and paused at the “TTTT” site, histones H3 and H4 have completely dissociated, leaving only the naked DNA.

**Figure 3:**
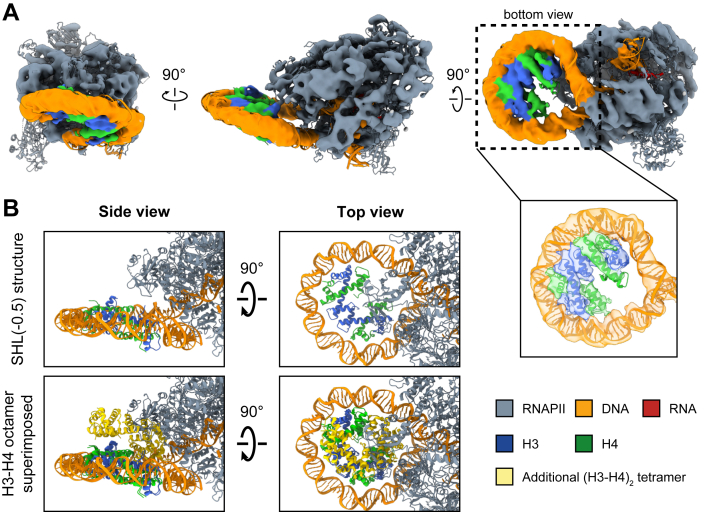
**Cryo-EM structure of the RNAPII–H3–H4 tetrasome (SHL[-0.5]).***A,* three different views of the same cryo-EM density map (with the structure model fitted) are shown. One (H3–H4)_2_ tetramer is retained in the structure, resulting in an H3–H4 tetrasome. A close-up view of the H3–H4 tetrasome region demonstrates that the α-helices of H3 and H4 are visualized in the H3–H4 tetrasome. *B,* the H3–H4 octamer, taken from a cryo-EM structure of the H3–H4 octasome (Protein Data Bank ID: 7X57), is superimposed onto the RNAPII–H3–H4 tetrasome (SHL[-0.5]) structure. The structures were aligned using the common (H3–H4)_2_ tetramer. The additional (H3–H4)_2_ tetramer, colored *yellow*, shows severe steric clashes with RNAPII. RNAPII, RNA polymerase II.

In the SHL(-0.5) structure, RNAPII is paused five base pairs before the dyad SHL(0) position (Fig. 3*A*). The resolution of this structure is limited as compared with the SHL(-4) structure, possibly because of the small number of particles used in the final reconstruction. Strikingly, we noticed that only one (H3–H4)_2_ tetramer is present in the SHL(-0.5) structure (Fig. 3*A*). This result suggests that an (H3–H4)_2_ tetramer may have dissociated from the H3–H4 octasome after RNAPII surmounted the SHL(-4) barrier but before it reached the SHL(-0.5) position.

To assess the outcome if the dissociation event did not occur, we superimposed a histone (H3–H4)_4_ octamer onto the current SHL(-0.5) structure (Fig. 3*B*). We found that if the (H3–H4)_2_ tetramer remained associated, it would clash severely with RNAPII. This shows that the dissociation of the proximal (H3–H4)_2_ tetramer is required for RNAPII to reach SHL(-0.5) of the H3–H4 octasome and that the dissociation may be caused by RNAPII.

**Figure 4:**
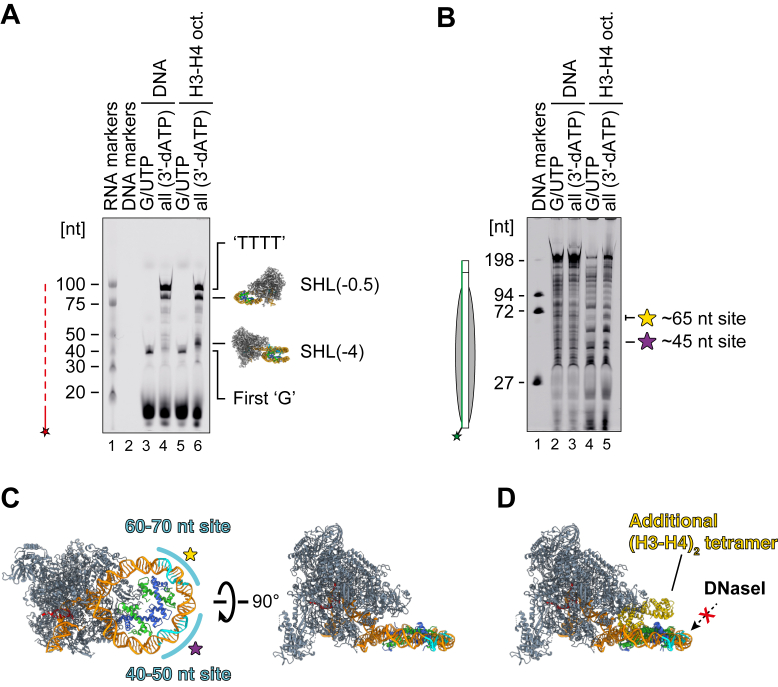
**DNase I footprinting analysis of the H3–H4 octasome transcribed by the Spt4/5–Elf1–RNAPII elongation complex.***A,* DY647 signals from the RNA products were detected, indicating the transcriptional progression of RNAPII on the template. *B,* 6-FAM signals from the DNA were detected, revealing the DNase I digestion pattern under each condition. Two sites showing intensified bands in lane 5 compared with lane 4 are marked with *stars*. *C,* these two sites were mapped back onto the RNAPII–H3–H4 tetrasome structure, with the +5 nt and −5 nt positions colored *cyan*. *D,* an additional (H3–H4)_2_ tetramer, taken from the cryo-EM structure of the H3–H4 octasome, is superimposed onto the RNAPII-H3–H4 tetrasome (SHL[-0.5]) structure and aligned using the common (H3–H4)_2_ tetramer. This highlights the potential inhibition of DNase I by the additional tetramer. The assay was repeated three times independently, and consistent results were obtained (Fig. S8). 6-FAM, 6-carboxyfluorescein; RNAPII, RNA polymerase II.

To further obtain biochemical evidence for the dissociation of the (H3–H4)_2_ tetramer, we also performed a DNase I footprinting analysis on the transcribed H3–H4 octasome. To this end, we prepared an H3–H4 octasome template for transcription with a 6-carboxyfluorescein (6-FAM) label at the distal end of the template strand (Figs. S7 and S8*A*). To enhance the processivity of RNAPII, we conducted the transcription reaction in the presence of elongation factors, Spt4/5 and Elf1. By including different NTPs in the reaction mixture, we could pause RNAPII at different positions on the template: either before it reaches the SHL(-4) position or near the SHL(-0.5) position (Figs. 4*A* and S8, *A*, *B*, and *D*). By comparing the DNase I digestion patterns, we identified two intensified bands in the more advanced RNAPII (Fig. 4*B*, lanes 4 and 5, and S8, C and E). These two bands roughly correspond to the 45 nt and 65 nt sites, which are located at the outer rim of the H3–H4 tetrasome, facing opposite RNAPII (Fig. 4*C*). These observations are consistent with the idea that an (H3–H4)_2_ tetramer may dissociate when RNAPII reaches the SHL(-0.5) position, as these sites are not expected to be accessible to DNase I in the presence of an additional (H3–H4)_2_ tetramer (Fig. 4*D*).

Because the remaining H3–H4 tetrasome may be relatively unstable, this could explain why RNAPII does not pause after overcoming the natural barrier at the SHL(-4) position. Therefore, the two (H3–H4)_2_ tetramer units may be dissociated in a stepwise manner from the H3–H4 octasome during RNAPII passage.

## Discussion

**Figure 5:**
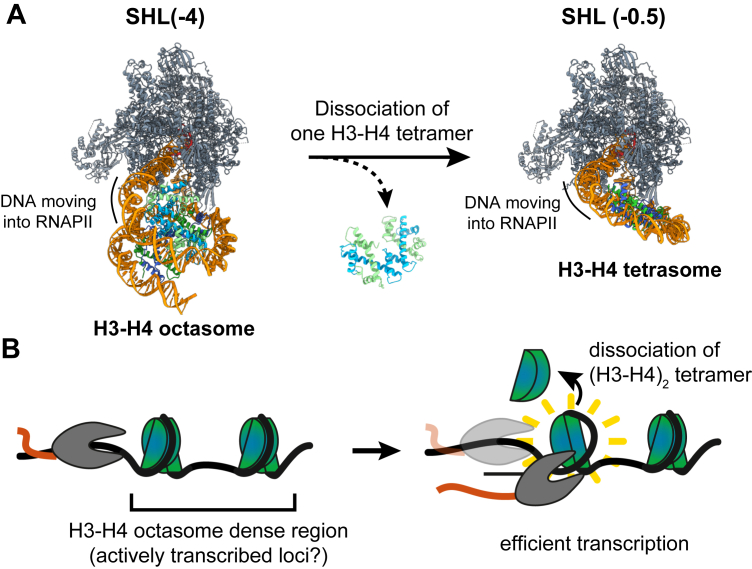
**Model of transcription on the H3–H4 octasome.***A,* snapshots of the transcription process of the H3–H4 octasome. When RNAPII encounters the H3–H4 octasome SHL(-4) (*left panel*), it pauses because of the DNA–histone interactions within the H3–H4 octasome and the interactions between RNAPII subunits and the H3–H4 octasome. As RNAPII overcomes the SHL(-4) barrier, one (H3–H4)_2_ tetramer dissociates from the H3–H4 octasome, leaving an H3–H4 tetrasome (*right panel*). This H3–H4 tetrasome is structurally unstable and therefore incapable of stopping RNAPII. *B,* in contrast to the nucleosomal transcription process, in which RNAPII pauses at SHL(-5), SHL(-2), and SHL(-1), in the H3–H4 octasomal transcription process, RNAPII only pauses at SHL(-4). This suggests that H3–H4 octasomes may be transcribed more efficiently than nucleosomes. RNAPII, RNA polymerase II; SHL, superhelical location.

We previously reported that the H3–H4 octasome wraps the DNA differently from the nucleosome (25). Although the H3–H4 octasome has been suggested to exist in cells, its function has remained elusive. In this study, we found that the transcription profile of the H3–H4 octasome differs from that of the nucleosome (Fig. 1*F*). During nucleosome transcription, RNAPII pauses at several SHLs, including SHL(-6), SHL(-5), SHL(-2), and SHL(-1). These nucleosome barriers of RNAPII transcription are drastically reduced by elongation factors, such as Spt4/5 and Elf1, and the nucleosome is eventually transferred to the upstream DNA region with the aid of other elongation factors and the histone chaperone FACT (Facilitates Chromatin Transcription) (10, 15). In contrast, RNAPII transcription of the H3–H4 octasome pauses only at SHL(-4). After overcoming the SHL(-4) barrier, RNAPII does not pause and finishes transcribing the remaining template (Fig. 1*F* and Movie S1). Given that RNAPII alone pauses at multiple positions in the nucleosome, the H3–H4 octasome may lead to more efficient transcription elongation than the nucleosome (Fig. 5).

Nevertheless, conserved mechanisms of RNAPII pausing exist between the H3–H4 octasome and the nucleosome. The strong histone–DNA interactions within the H3–H4 octasome at the SHL(-4) position likely contribute to the natural RNAPII pausing (Fig. 2). In the nucleosome, this histone–DNA interaction of the H3–H4 octasome is conserved in the SHL(-1) position, which also induces strong natural RNAPII pausing (8). In the nucleosome SHL(-1) position, the H3R63, H3K64, H3R69, and H4R36 residues interact with DNA, forming an obstacle for RNAPII to peel off the DNA. These histone residues also contribute to the SHL(-4) pausing in the H3–H4 octasome (Fig. 2*B*), suggesting that the pausing mechanism induced by histone–DNA interactions may be conserved between the nucleosome and the H3–H4 octasome and possibly also among other noncanonical nucleosomes. However, more investigation is required to determine whether the histone–DNA interaction is a universal mechanism in transcription pausing.

Our cryo-EM analyses revealed that, in the H3–H4 octasome, the (H3–H4)_2_ tetramers are dissociated by RNAPII progression in a stepwise manner during transcription. This is reminiscent of the stepwise histone eviction observed in nucleosomes containing the histone variant H2A.B (29). In our *K. phaffii* RNAPII experimental system, all histones are retained when RNAPII reaches the SHL(0) position during transcription in the canonical nucleosome (8, 10, 15), although H2A–H2B eviction has been reported in certain mammalian systems (13). Therefore, noncanonical nucleosomes, such as the H3–H4 octasome and H2A.B-containing nucleosomes, may facilitate transcription elongation by promoting histone dissociation.

In summary, the present study reveals how a recently reported atypical nucleosome, the H3–H4 octasome, is transcribed by RNAPII. The fact that RNAPII only pauses at one site, SHL(-4), suggests that the H3–H4 octasome may be transcribed more efficiently than the nucleosome. Therefore, the H3–H4 octasome may exist in highly transcribed genomic loci. Remaining issues to understand are as follows: (i) How do transcription elongation factors, such as Spt4/5 and Elf1, affect the transcription of the H3–H4 octasome? (ii) Is the H3–H4 octasome reassembled or discarded after the RNAPII passes through its dyad DNA region? and (iii) Where do the genomic loci enriched in H3–H4 predominantly exist and function? The H3–H4 octasome lacks the acidic patch, a region in the nucleosome where various chromatin-interacting factors, such as histone chaperones, nucleosome remodelers, and histone modifiers, typically bind. Therefore, the lack of an acidic patch in the H3–H4 octasome may have distinct functions in genome regulation from the canonical nucleosome. It should also be noted that our experiments have been performed with a strong nucleosome-positioning DNA sequence. RNAPII may traverse the H3–H4 octasome more efficiently in a natural DNA sequence context. Further studies will be required to solve these issues.

## Experimental procedures

### Purification of proteins

Human histones H3.1 and H4 were prepared as previously described (30). Briefly, N-terminally hexa-histidine (His_6_)-tagged H3.1 was expressed in *Escherichia coli* BL21 (DE3) cells, and N-terminally His_6_-tagged H4 was expressed in *E. coli* JM109 (DE3) cells. The *E. coli* cells were then sonicated, and the lysates were denatured. Under denaturing conditions, the proteins were purified by nickel–nitrilotriacetic acid agarose resin (QIAGEN) chromatography. The His_6_-tag was removed by thrombin protease under nondenaturing conditions. Finally, the histones were purified by cation exchange chromatography under denaturing conditions on a Mono S column. The collected histone fractions were dialyzed against water and lyophilized. RNAPII was purified from the yeast *K. phaffii*, and TFIIS, Spt4, Spt5, and Elf1 were purified as recombinant proteins as previously described (28).

### Preparation of DNA fragments for H3–H4 octasome reconstitution

Three kinds of 153 base pair DNA fragments were prepared for reconstituting H3–H4 octasomes.

To reconstitute the H3–H4 octasome initially used in the transcription assays, the DNA fragment (unmodified) was prepared as previously described (8). Briefly, the plasmid DNA containing a modified version of the Widom 601 sequence (31) was amplified in *E. coli*. The plasmid DNA was then collected and digested by EcoRV to obtain the target DNA fragment. After purification by polyethylene glycol precipitation, the DNA fragment was dephosphorylated to prevent self-ligation in the following steps. Finally, the DNA fragment was cleaved by BglI (Takara) and purified by DEAE-5PW anion-exchange column chromatography (TOSOH). The sequence is identical to the previously used DNA fragment (8).

To reconstitute the modified H3–H4 octasome used in the subsequent transcription assays and cryo-EM analysis, the DNA fragment (modified) was prepared by PCR. The modified DNA fragment is similar to the unmodified version, with only “ATT” substituted with “TAA” in the template strand (the resulting sequence is shown at the end of the paragraph). After PCR, the DNA fragment was then dephosphorylated and cleaved by BglI. Finally, using a Prep Cell apparatus (Bio-Rad), the DNA was purified by nondenaturing PAGE. The resulting sequence is as follows, with the modified locations shown in bold letters:

Template strand: 5′-ATCAG AATCC CGGTG CCGAG GCCGC TCAAT TGGTC GTAGA CAGCT CTAGC ACCGC TTAAA CGCAC GTACG CGCTG TCCCC CGCGT TTTAA CCGCC AAGGG G**TAA**A CACCC AAGAC ACCAG GCACG AGACA GAAAA AAACA ACGAA AACGG CCACC A-3′; Nontemplate strand: 5′-TGGCC GTTTT CGTTG TTTTT TTCTG TCTCG TGCCT GGTGT CTTGG GTGT**T TA**CCC CTTGG CGGTT AAAAC GCGGG GGACA GCGCG TACGT GCGTT TAAGC GGTGC TAGAG CTGTC TACGA CCAAT TGAGC GGCCT CGGCA CCGGG ATTCT GAT-3′.

For the DNase I footprinting experiment, the modified DNA was amplified using a forward primer bearing a 5′-terminal 6-FAM label, such that the fluorescent tag is positioned at the 5′ end of the template strand (preceding the ATCAG sequence). The subsequent BglI treatment and purification procedures are identical.

### Preparation of the template H3–H4 octasomes containing nine base-mismatched DNA regions

The template H3–H4 octasome for transcription was prepared in a two-step method, similar to the previously reported method for preparing the nucleosome template (8).

First, using the salt dialysis method, H3–H4 octasomes were reconstituted with one of the 153 bp DNA fragments described in the previous section (unmodified or modified) and the (H3–H4)_2_ tetramer, which was refolded and purified as described previously (25). The H3–H4 octasomes were heated and purified by nondenaturing PAGE, using a Prep Cell apparatus (25).

Second, the short DNA fragment with a nine-base mismatch was ligated to the H3–H4 octasome by T4 DNA ligase (NIPPON GENE). The DNA sequence is identical to the one used in the previous study (8). After ligation, the H3–H4 octasomes were purified one final time by nondenaturing PAGE, using a Prep Cell apparatus.

The H3–H4 octasome used in the DNase I footprinting assay was further dialyzed against a buffer containing 5% glycerol (20 mM Tris–HCl [pH 7.5], 1 mM DTT, and 5% glycerol), flash-frozen, and stored at −80 °C until use.

### Transcription assay on the H3–H4 octasomes

*In vitro* transcription assays using the unmodified template were performed by adding 0.1 μM RNAPII, 0.1 μM TFIIS, and 0.4 μM primer RNA (5′-DY647-AUAAUUAGCUC-3′) (Dharmacon) to the reaction solution (26 mM Hepes–KOH [pH 7.5], 5 mM MgCl_2_, 50 mM potassium acetate, 0.3 μM zinc acetate, 33 μM Tris(2-carboxyethyl)phosphine [TCEP], 1.6% glycerol, 400 μM UTP, 400 μM CTP, 400 μM GTP, and 400 μM ATP). To start the transcription reaction, 0.15 μM (final concentration) of template DNA, template nucleosome, or template H3–H4 octasome was added to the reaction solution, in a final volume of 15 μl. The solution was then incubated at 30°C for 5 min. Subsequently, 4 μl of the reaction solution was collected and mixed with 2 μl of ProK solution (20 mM Tris [pH 7.5], 20 mM EDTA, and 446 μg/μl proteinase K) to terminate the reaction. To denature the RNA product, 24 μl of Hi-Di formamide (Applied Biosystems) was added, and the solution was incubated at 95 °C for 5 min. Finally, the RNA products were fractionated by 10% denaturing PAGE and detected by DY647 fluorescence, using an Amersham Typhoon scanner (Cytiva). DynaMarker DIG–labeled Blue Color Marker for Small RNA (BioDynamics Laboratory) served as the standard RNA markers.

*In vitro* transcription assays using the modified template were performed by mixing 0.225 μM naked DNA or H3–H4 octasome, 0.4 μM RNAPII, 0.15 μM TFIIS, and 0.6 μM primer RNA (5′-DY647-AUAAUUAGCUC-3′) (Dharmacon) in 10 μl of reaction solution (24 mM Hepes–KOH [pH 7.5], 5 mM MgCl_2_, 30 mM potassium acetate, 0.2 μM zinc acetate, 20 μM Tris(2-carboxyethyl)phosphine, 1% glycerol, 400 μM UTP, 400 μM CTP, 400 μM GTP, and 400 μM ATP or 40 μM 3′-dATP). The solution was then incubated at 30 °C. At the indicated times, 1 μl of the reaction solution was removed and mixed with 1 μl of ProK solution (20 mM Tris [pH 7.5], 20 mM EDTA, and 446 μg/μl proteinase K) to terminate the reaction. To denature the RNA product, 24 μl of Hi-Di formamide (Applied Biosystems) was added, and the solution was incubated at 95 °C for 10 min. Finally, the RNA products were fractionated by 10% denaturing PAGE and detected by DY647 fluorescence, using an Amersham Typhoon scanner (Cytiva). DynaMarker DIG–labeled Blue Color Marker for Small RNA (BioDynamics Laboratory) served as the standard RNA markers.

### Preparation of the RNAPII–H3–H4 octasome–tetrasome complexes for cryo-EM analysis

The transcription reaction was conducted by mixing 0.23 μM H3–H4 octasome, 0.40 μM RNAPII, 0.15 μM TFIIS, and 0.6 μM primer RNA (5′-DY647-AUAAUUAGCUC-3′) (Dharmacon) in 1000 μl of reaction solution (33 mM Hepes–KOH [pH 7.5], 3 mM Tris–HCl [pH 7.5], 5.0 mM MgCl_2_, 400 μM UTP, 400 μM CTP, 400 μM GTP, 400 μM ATP, 97 mM KOAC, 0.64 μM Zn(OAc)_2_, 64 μM TCEP–HCl, 0.15 mM DTT, and 3.2% glycerol). The solution was incubated at 30 °C for 40 min, and then 20 μl of 500 mM EDTA was added to terminate the reaction. The complex was further purified by the GraFix method (32), with a low buffer composed of 20 mM Hepes–KOH (pH 7.5), 50 mM KOAc, 0.2 μM Zn(OAc)_2,_ 0.5 mM TCEP–HCl, and 10% (w/v) sucrose, and a high buffer composed of 20 mM Hepes–KOH (pH 7.5), 50 mM KOAc, 0.2 μM Zn(OAc)_2,_ 0.5 mM TCEP–HCl, 25% (w/v) sucrose, and 0.1% glutaraldehyde. The reaction mixture was loaded onto the gradient, and the samples were centrifuged at 30,000 rpm and 4°C for 14 h, using a Beckman SW32 Ti rotor.

After centrifugation, 1 ml fractions were collected from the top of the gradient solution, using a pipette. To determine the range for collection, the fractions were analyzed by 4% nondenaturing PAGE with RNAPII–H3–H4 octasome–tetrasome complex detection by SYBR Gold, and 10% denaturing PAGE with elongated RNA product visualization by DY647 fluorescence, using an Amersham Typhoon Imager (GE Healthcare). Fractions containing the elongated RNAPII–H3–H4 octasome–tetrasome complexes were collected and then buffer exchanged on a PD10 column into 20 mM Hepes–NaOH (pH 7.5), 0.2 μM Zn(OAc)_2,_ and 0.1 mM TCEP–HCl. Finally, the sample was concentrated by an Amicon Ultra 100K filter to a final DNA concentration of 0.51 mg/ml.

For cryo-EM analysis, Quantifoil grids (R1.2/1.3 Cu 200 mesh, Quantifoil Micro Tools) were glow discharged for 1 min with a PIB-10 ION Bombarder (Vacuum Device, Inc), immediately before use. Subsequent processes were performed using a Vitrobot Mark IV (Thermo Fisher Scientific). Portions (2.5 μl) of diluted samples (DNA concentration 0.05 mg/ml) were applied to the grids, blotted for 4 or 5 s with a blot force of 5 at 4 °C/100% humidity, and plunge frozen.

### Cryo-EM data collection

Cryo-EM images were recorded by a Krios G4 transmission electron microscope (Thermo Fisher Scientific), equipped with a K3 direct electron detector (Gatan). Automated data acquisition was performed with the EPU software (pixel size: 1.06 Å, defocus values: between −1.0 and −2.5 μm, total dose: 59.2–60.8 e^−^/A^2^ for over 40 frames). In total, three datasets were collected. More information can be found in Table S1.

### Cryo-EM image processing

The cryo-EM images of each dataset were imported into Relion4 for single-particle analysis (33). Motion correction was performed by MotionCor2 (34), and contrast transfer function estimation was performed by CTFFIND4 (35). Subsequently, micrographs with poor quality were discarded by manual selection.

A subset of 1000 micrographs from the first dataset were then chosen for training a Topaz model for autopicking. In brief, autopicking was first performed in the Laplacian-of-Gaussian mode, and 2D classification was performed to discard junk particles. The remaining particles were used for training the first Topaz model.

This Topaz model was then used for autopicking particles from the entire first dataset (36). 2D and 3D classifications were performed to discard junk particles. The remaining particles were then used to train a second Topaz model, which was the main model used for autopicking particles from the first, second, and third datasets.

The particles from these different datasets were then merged and subjected to 2D and 3D classifications (Fig. S4). In the second round of 3D classification, two major classes of the particles can be observed. These two classes were selected separately, and all downstream analyses of the two classes were performed independently.

For the SHL(-4)-like particles, several additional rounds of 3D classification were performed in Relion to remove bad particles and eliminate the effect of orientation bias (Fig. S5). The remaining good particles were imported to CryoSPARC (37) for subsequent processing. To remove particles in which the H3–H4 octasome subunit was unclear, one extra round of 3D classification (without alignment) was performed in CryoSPARC with an H3–H4 octasome mask. Finally, Bayesian-polished particles of the chosen class were subjected to 3D flexible refinement. The output map of the 3D flexible refinement was sharpened for model building and is displayed in Figure 2.

For the SHL(-0.5)-like particles, one additional round of 3D classification was performed in Relion, before importing the selected particles into CryoSPARC (Fig. S6). At this stage, we noticed that severe orientation bias was present in the SHL(-0.5) particles, making it impossible to obtain a clear map of both the RNAPII and the H3–H4 tetrasome. Because the structure of RNAPII is not expected to differ much from the previously reported structures, we decided to focus mainly on the H3–H4 tetrasome instead, which is the highlight of this study. To visualize the histone helices of the H3–H4 tetrasome, two rounds of 3D classifications (without alignment) were performed in CryoSPARC with an H3–H4 tetrasome mask. A subsequent 3D classification (without alignment) was performed to slightly reduce the effect of orientation bias on the RNAPII. It should be noted that the multiple rounds of 3D classification, performed to mitigate orientation bias rather than particle heterogeneity, resulted in the rejection of a large number of particles. Finally, Bayesian-polished particles of the selected class were subjected to 3D flexible refinement. The output map of the 3D flexible refinement was sharpened for model building and is displayed in Figure 3. More information can be found in Table S1.

### Model building

Modeling for both the RNAPII–H3–H4 octasome and the RNAPII–H3–H4 tetrasome complex was based on the atomic model of the RNAPII–nucleosome complex stalled at SHL(-5) (Protein Data Bank [PDB] ID: 6A5P) (8). For the RNAPII–H3–H4 octasome complex, the nucleosome was first replaced by the H3–H4 octasome (closed form) atomic model (PDB ID: 7X57) (25). The edited atomic model was then refined using the phenix.real_space_refine tool in the Phenix package (38) and modeled manually to fix outliers by using the Coot software (39, 40). For the RNAPII–H3–H4 tetrasome complex, the nucleosome was replaced by half of the H3–H4 octasome (closed form) atomic model (PDB ID: 7X57) (25). Due to the limited resolution of the cryo-EM map, no further refinement was performed (only validation). More information can be found in Table S1.

### DNase I footprinting analysis

The DNase I footprinting analysis was conducted using a similar scheme to that previously reported (29). Briefly, the 6-FAM-labeled DNA or H3–H4 octasome was first transcribed by RNAPII, followed by DNase I digestion, proteinase K treatment, and analysis by denaturing PAGE.

The transcription reaction was carried out by mixing the 6-FAM-labeled DNA–H3–H4 octasome (0.23 μM), 0.4 μM RNAPII, 0.4 μM TFIIS, 1.6 μM Spt4/5, 4.0 μM Elf1, and 0.3 μM primer RNA (5′-DY647-AUAAUUAGCUC-3′) (Dharmacon) in the reaction solution (8 μl), which contained 3 mM Tris–HCl (pH 7.5), 35 mM Hepes–KOH (pH 7.5), 5 mM MgCl_2,_ 0.15 mM DTT, 113 mM potassium acetate, 0.75 μM zinc acetate, 75 μM TCEP, 4.5% glycerol, and either GTP/UTP (0.4 mM each) or GTP/UTP/CTP/3′-dATP (0.4 mM each). By including only GTP and UTP in the reaction mixture, RNAPII stops immediately before entering the H3–H4 octasome. On the other hand, by including 3′-dATP, UTP, CTP, and GTP in the reaction mixture, RNAPII progresses further on the template and stops at the engineered “T” and “TTTT” sites near SHL (−0.5) of the H3–H4 octasome. The transcription reaction was carried out at 30 °C for 40 min.

Subsequently, 2 μl of DNase I (Takara) was added to the reaction mixture; 0.0018 units were added to the DNA samples, whereas 0.0180 units were added to the H3–H4 octasome samples. The digestion reaction was carried out at 30 °C for 10 min, before being terminated by adding 5 μl of ProK solution (20 mM Tris [pH 7.5], 20 mM EDTA, 446 μg/μl proteinase K [Roche]). Then, 7.5 μl aliquots of these mixtures were collected and mixed with 30 μl of Hi-Di formamide (Thermo Fisher Scientific), incubated at 95 °C for 10 min, and analyzed by 10% denaturing PAGE. DY647 and 6-FAM fluorescence signals were detected by using an Amersham Typhoon scanner (Cytiva). DynaMarker DIG–labeled Blue Color Marker for Small RNA (BioDynamics Laboratory) served as the standard RNA markers. The DNA markers were generated from the 198 bp 6-FAM template DNA by digesting it with either of the following restriction enzymes: Mfe-HF (NEB), HhaI (Takara), or StyI-HF (NEB). The lengths of the digested DNA were estimated by ImageQuant (Cytiva).

### Data availability

The data that support this study are available from the corresponding authors upon reasonable request. The cryo-EM reconstructions and atomic models of the RNAPII–H3–H4 octasome complex and the RNAPII–H3–H4 tetrasome complex generated in this study have been deposited in the Electron Microscopy Data Bank and the PDB, under the accession codes EMD-64225 and PDB ID 9UJS, and EMD-64226 and PDB ID 9UJT, respectively.

## Supporting information

This article contains supporting information.

## Declaration of generative AI and AI-assisted technologies in the writing process

ChatGPT was used for grammatical correction, rephrasing and/or rearranging parts of the text. No completely original content was produced by AI. The authors take full responsibility for the content of the article, and they have reviewed the outputs and edited them as necessary.

## Conflict of interest

The authors declare that they have no conflicts of interest with the contents of this article.
