# Supplementary material for: Structural basis of RNA polymerase II transcription on the histone H3–H4 octasome
Source: J Biol Chem. 2026 Mar 4;302(4):111340. doi: 10.1016/j.jbc.2026.111340 (PMC13085070; doi:10.1016/j.jbc.2026.111340)
Supplement: Figures S1-S8 and Table S1 [file mmc2.docx]

**Supporting Information**

Structural basis of RNA polymerase II transcription on the histone H3-H4 octasome

Cheng-Han Ho（何　承翰）^1^, Kayo Nozawa（野澤　佳世）^1^, Masahiro Nishimura（西村　正宏）^1^, Mayuko Oi（大井　茉祐子）^1,2^, Tomoya Kujirai（鯨井　智也）^1,3^, Mitsuo Ogasawara（小笠原　光雄）^1^, Haruhiko Ehara（江原　晴彦）^3^, Shun-ichi Sekine（関根　俊一）^3^, Yoshimasa Takizawa（滝沢　由政）^1^, and Hitoshi Kurumizaka（胡桃坂　仁志）^1,2,3^*

^1^ Laboratory of Chromatin Structure and Function, Institute for Quantitative Biosciences, The University of Tokyo, 1-1-1 Yayoi, Bunkyo-ku, Tokyo 113-0032, Japan.

^2^ Department of Biological Sciences, Graduate School of Science, The University of Tokyo, 1-1-1 Yayoi, Bunkyo-ku, Tokyo 113-0032, Japan.

^3^ Laboratory for Transcription Structural Biology, RIKEN Center for Biosystems Dynamics Research, 1-7-22 Suehiro-cho, Tsurumi-ku, Yokohama 230-0045, Japan.

* To whom correspondence should be addressed. Tel: +81-3-5841-7826; Fax: +81-3-5841-1468; Email: kurumizaka@iqb.u-tokyo.ac.jp

Present Address:

Kayo Nozawa, School of Life Science and Technology, Tokyo Institute of Technology, 4259 Nagatsuta-cho, Midori-ku, Yokohama, Kanagawa 226-8501, Japan

Masahiro Nishimura, Epigenetics and Stem Cell Biology Laboratory, National Institute of Environmental Health Sciences, Research Triangle Park, NC, 27709, USA

**This PDF file includes: Figures S1-8, Table S1, Movie S1 (legend)**

**
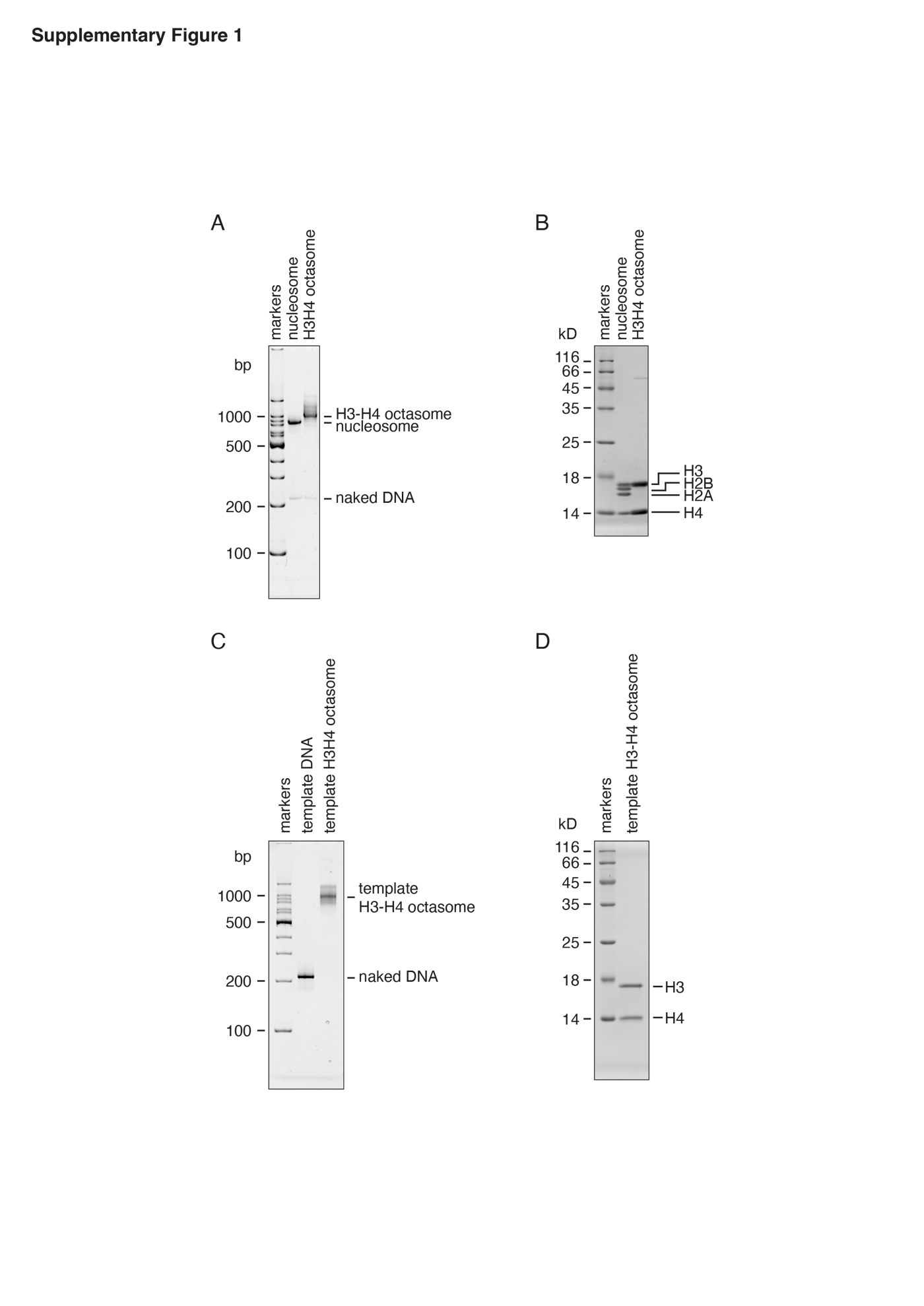
**

**Figure S1: H3-H4 octasome sample preparations.** (A) Purified nucleosome and H3-H4 octasome (reconstituted with the unmodified DNA) were analyzed by native polyacrylamide gel electrophoresis (PAGE) and visualized by ethidium bromide (EtBr) staining to detect DNA. (B) The same samples from (A) were also analyzed by SDS-PAGE and visualized by Coomassie Brilliant Blue (CBB) staining to detect proteins. (C) Purified template DNA and H3-H4 octasome (reconstituted with the modified template DNA) were analyzed by native-PAGE and visualized by EtBr staining to detect DNA. (D) The H3-H4 octasome from (C) was also analyzed by SDS-PAGE and visualized by CBB staining to detect proteins.

**
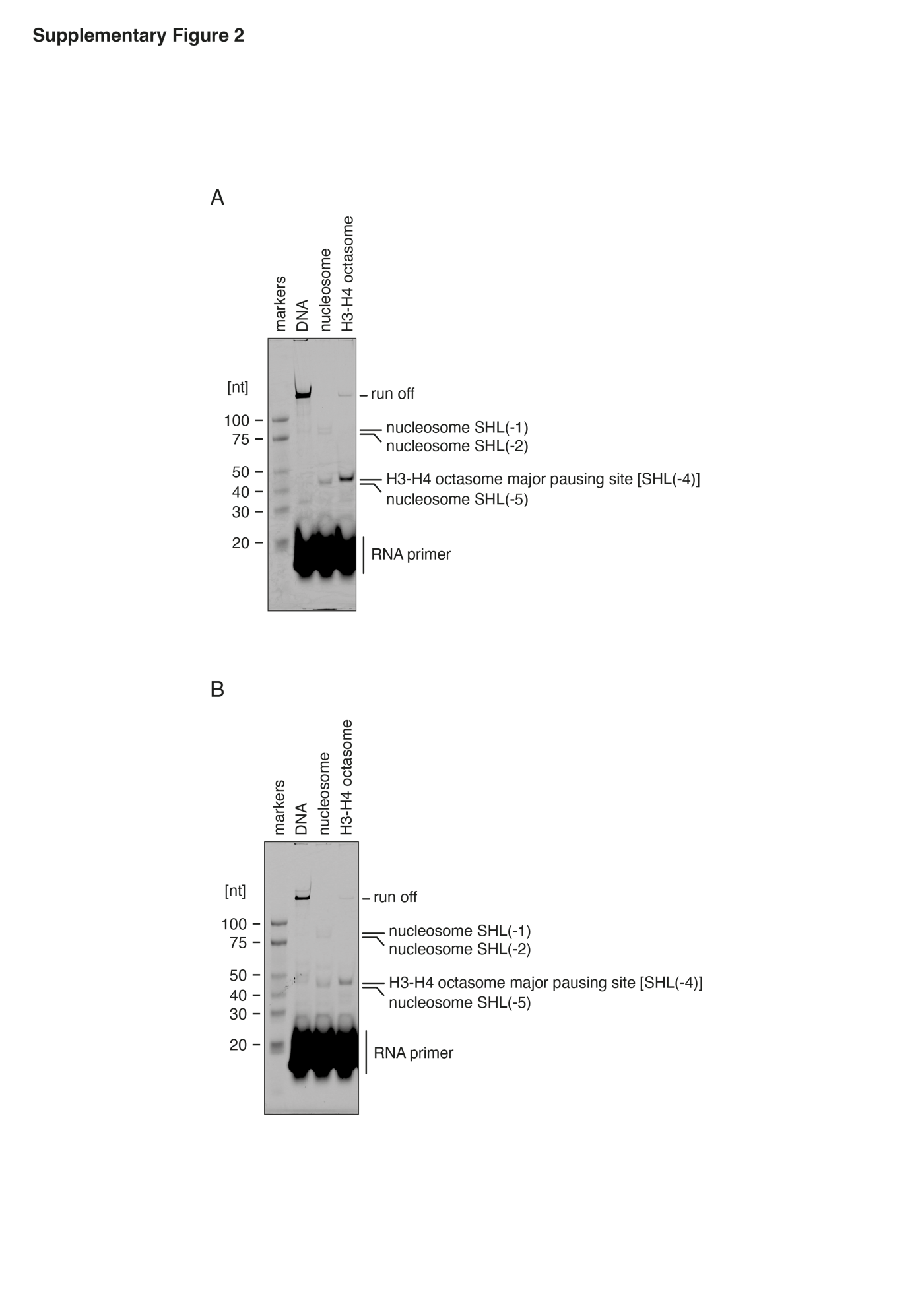
**

**Figure S2: Transcription assay of the H3-H4 octasome using unmodified DNA template.** The transcription assay in Fig. 1f was repeated three times independently in total. The 2 repeats other than Fig. 1F is shown in (A) and (B).


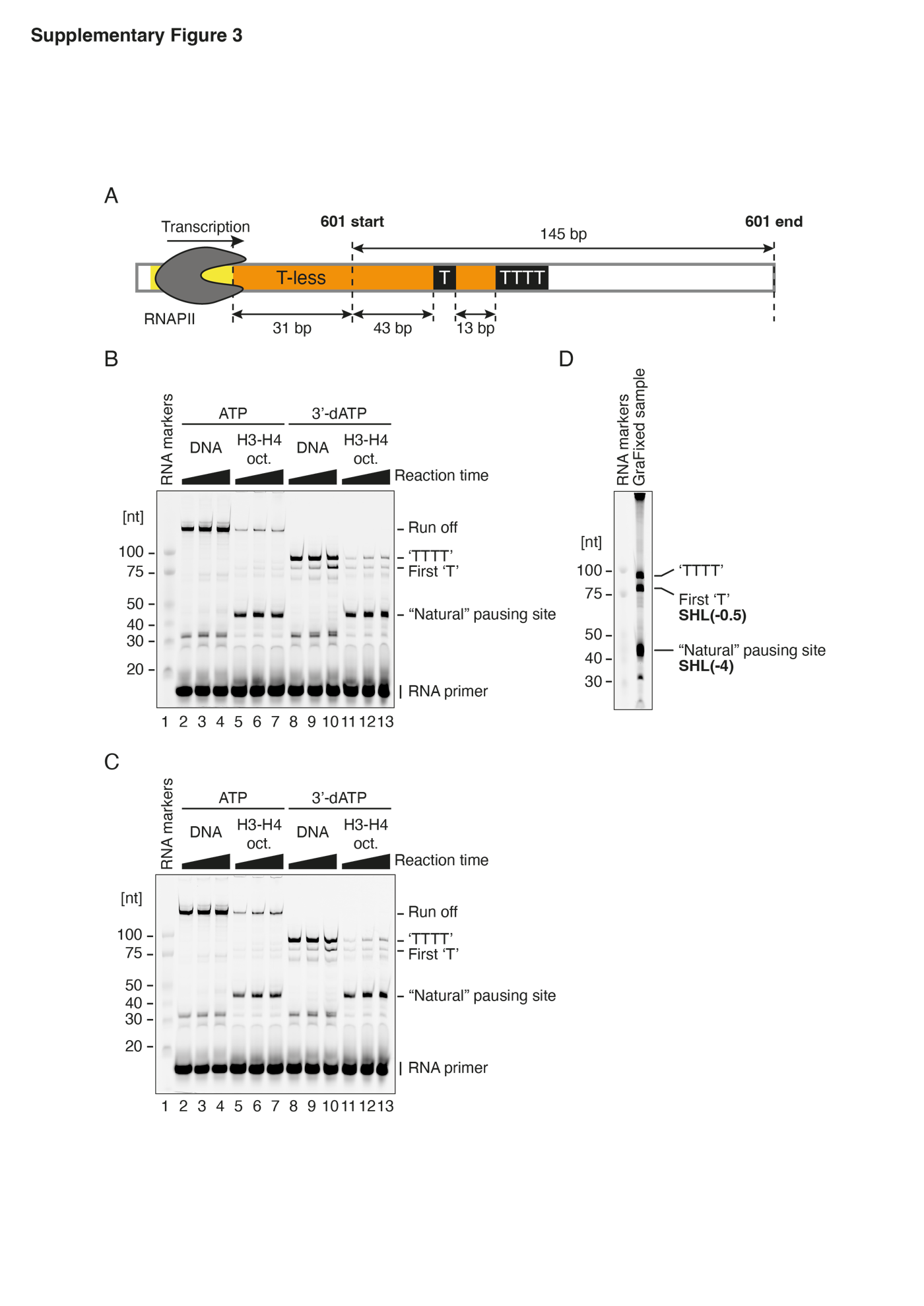


**Figure S3: Transcription assay of the H3-H4 octasome using modified DNA template.** (A) The modified DNA template. A new ‘T’ site is added 74 bp downstream of the transcription start site. The original ‘TTTT’ site also exists 13 bp downstream of the inserted ‘T’ site. (B, C) Transcription assays using the modified DNA template. The reaction times were 5, 20, and 40 minutes. ATP or 3 ́-dATP was used as indicated. (D) The GraFix-processed RNAPII-H3-H4 octasome sample was analyzed by denaturing gel electrophoresis. The RNA products were detected.


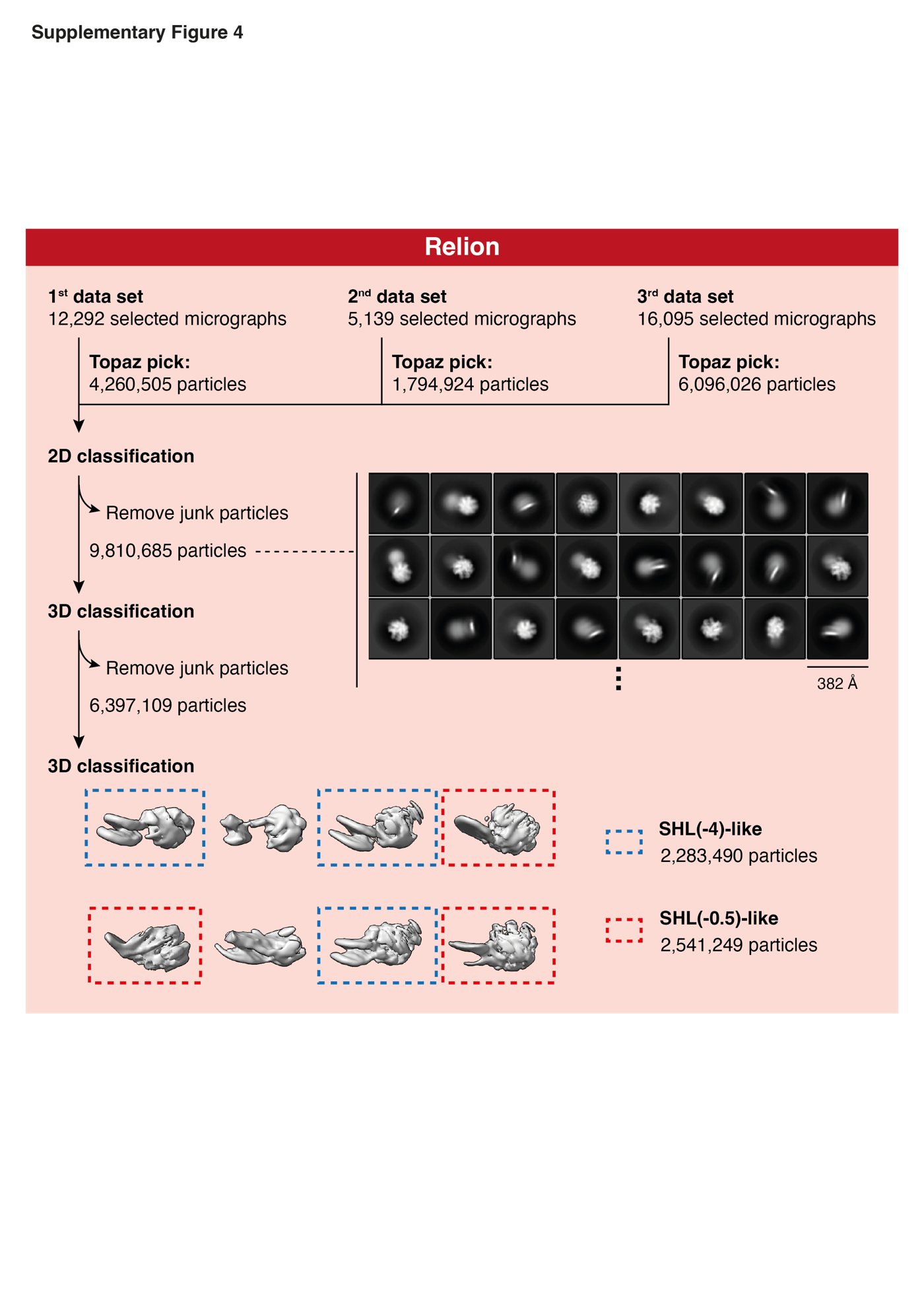


**Figure S4: Cryo-EM single particle analysis of data sets by Relion.** Three data sets were obtained and analyzed independently before 2D classification. After Topaz picking, the particles were merged and subjected to 2D classification. A subset of the 2D classes is shown. After two rounds of 3D classification, SHL(-4)-like particles and SHL(-0.5)-like particles were separated and subsequently processed individually.


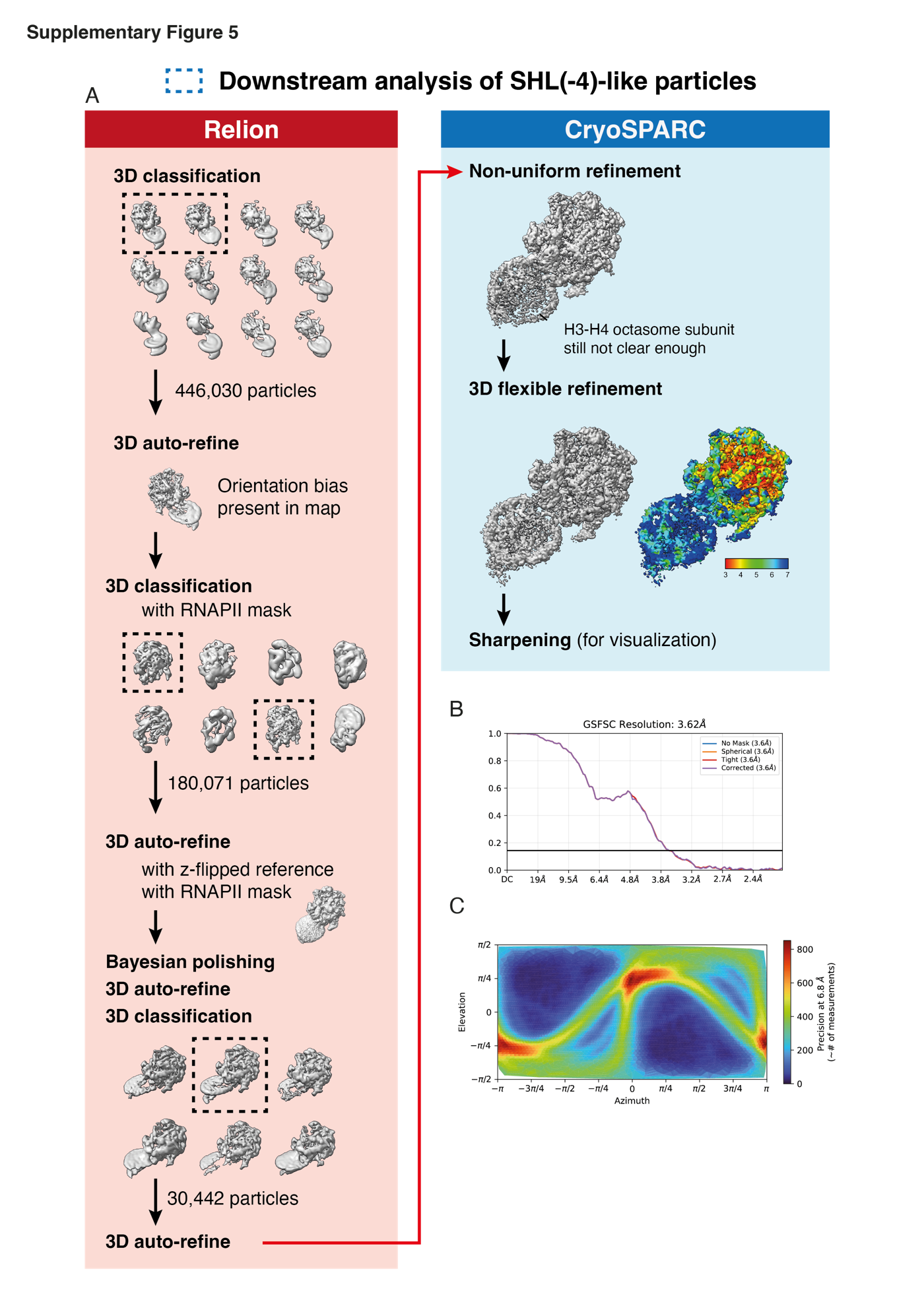


**Figure S5: Cryo-EM single particle analysis of the SHL(-4)-like particles.** (A) The SHL(-4)-like particles from Supplementary Figure 4 were further processed by both Relion and CryoSPARC. (B) Fourier shell correlation curves (C) Angular distributions


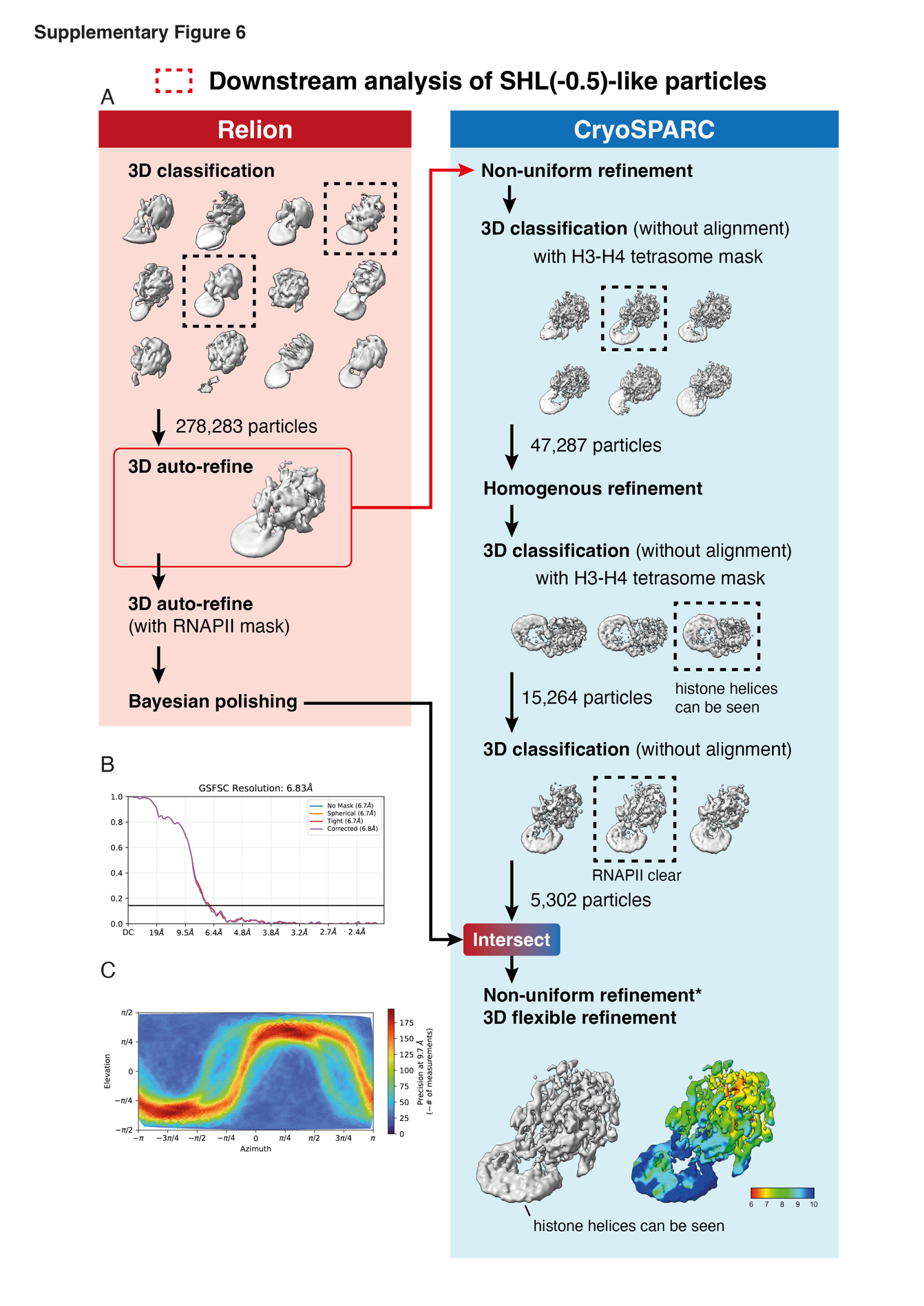


**Figure S6. Cryo-EM single particle analysis of the SHL(-0.5)-like particles.** (A) The SHL(-0.5)-like particles from Supplementary Figure 4 were further processed by both Relion and CryoSPARC. (B) Fourier shell correlation curves (C) Angular distributions


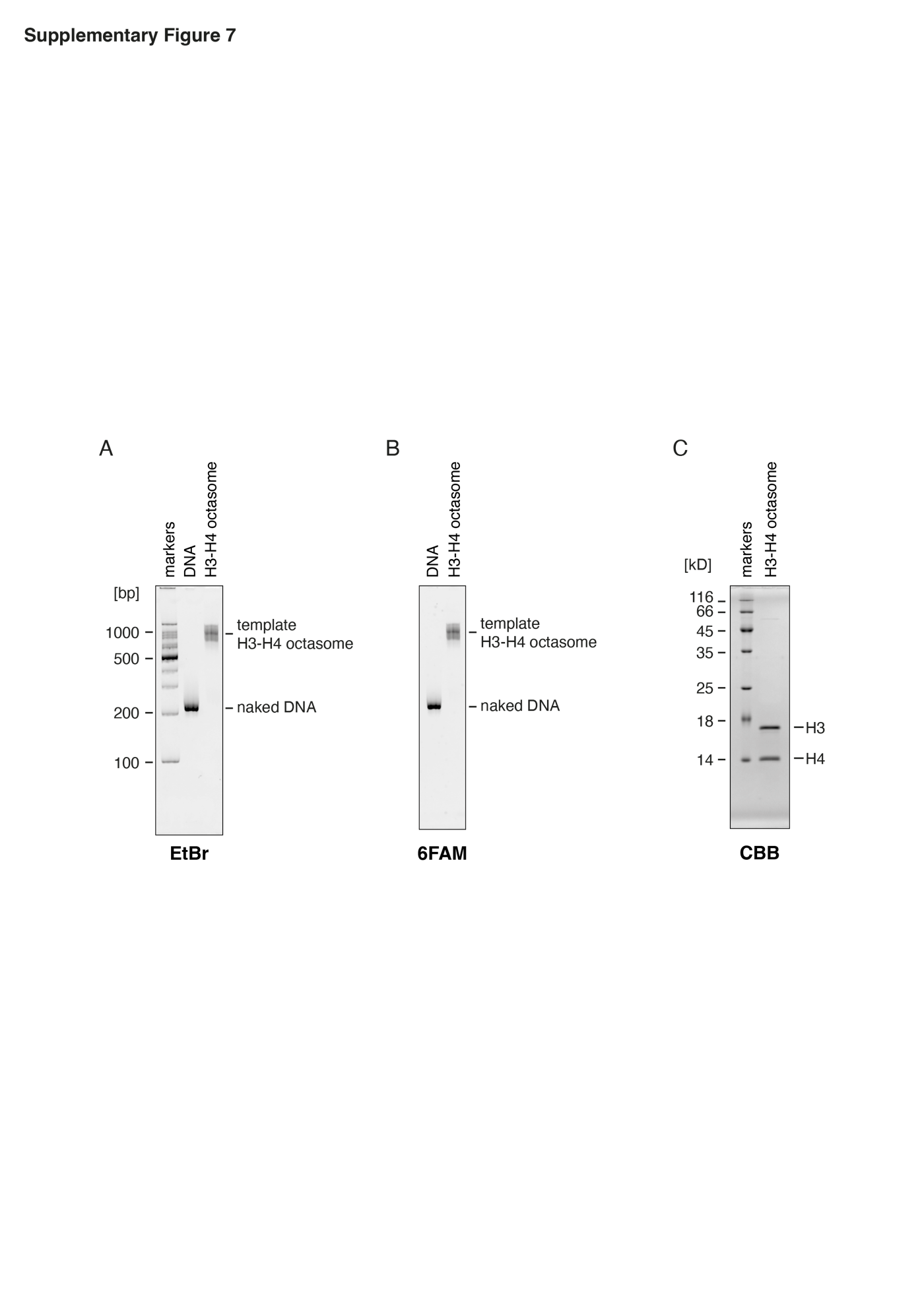


**Figure S7. 6-FAM-labelled H3-H4 octasome sample preparations.** (A) The 6-FAM-labelled DNA and H3-H4 octasome were analyzed by native polyacrylamide gel electrophoresis (PAGE) and visualized by ethidium bromide (EtBr) staining to detect DNA. (B) The gel from (A) was also detected by 6FAM to confirm proper labelling. (C) The same H3-H4 octasome from was also analyzed by SDS-PAGE and visualized by Coomassie Brilliant Blue (CBB) staining to detect proteins.


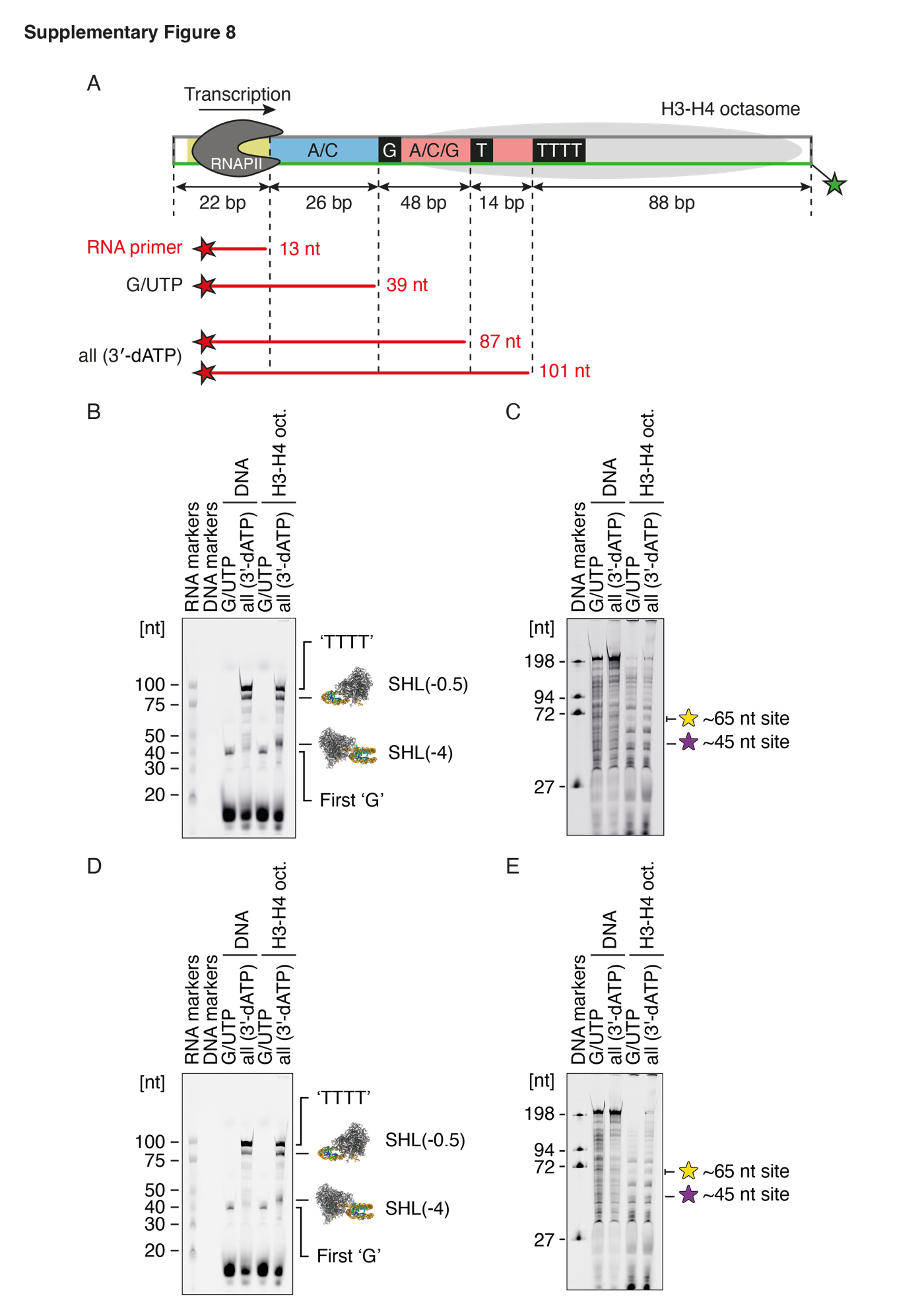


**Figure S8. DNase I footprinting analysis of the H3-H4 octasome transcribed by the Spt4/5-Elf1-RNAPII elongation complex.** (A) Design scheme of the template DNA. (B-E) Repeats of Fig. 4. (B, D) DY647 signals from the RNA products were detected, indicating the transcriptional progression of RNAPII on the template. (C,E) 6-FAM signals from the DNA were detected, revealing the DNase I digestion pattern under each condition. Two sites showing intensified bands in lane 5 compared with lane 4 are marked with stars.

**Table S1. Cryo-EM data collection and image processing**

**Movie S1. Transcription process of the H3-H4 octasome**
